# Ethanol(nitrato)[tris­(4-cyano-3-phenyl-1*H*-pyrazol-1-yl)hydroborato]nickel(II)

**DOI:** 10.1107/S2414314621006908

**Published:** 2021-07-09

**Authors:** Elvin V. Salerno, Lava R. Kadel, David M. Eichhorn

**Affiliations:** a Department of Chemistry and Biochemistry, Wichita State University, 1845 Fairmount St., Wichita, KS 67260-0051, USA; Howard University, USA

**Keywords:** crystal structure, nickel complex, scorpionate ligand

## Abstract

The synthesis and structure is reported of Tp^Ph,4CN^Ni(NO_3_)(EtOH) or [Ni(C_30_H_19_BN_9_)(NO_3_)(C_2_H_6_O)], the first half-sandwich complex of a cyano­scorpionate ligand. The pseudo­octa­hedral coordination sphere of the Ni^II^ ion is comprised of a tridentate tris­(4-cyano-3-phenyl­pyrazol­yl)borate ligand, a bidentate nitrate ligand and a neutral ethanol ligand. The phenyl substituents on the Tp^Ph,4CN^ ligand are relatively parallel to the planes of the ethanol and nitrate ligands. An inter­molecular hydrogen-bonding inter­action is evident between the ethanol OH group and the pyrazole CN substituent. The ethanol ligand was modeled with a 0.572 (13)/0.428 (13) disorder of the methyl C atom.

## Abstract 2

The synthesis and structure are reported of Tp^Ph,4CN^Ni(NO_3_)(EtOH), an octa­hedral complex containing a tridentate cyano­scorpionate ligand, a bidentate nitrate ligand and a neutral ethanol ligand.

## Graphical abstract

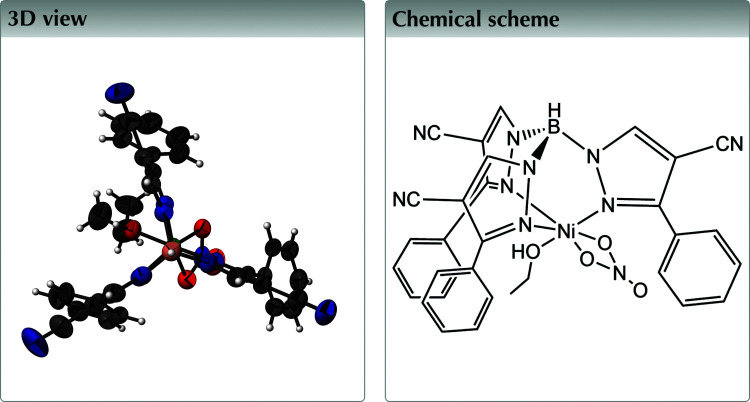

## Structure description

**Figure 1:**
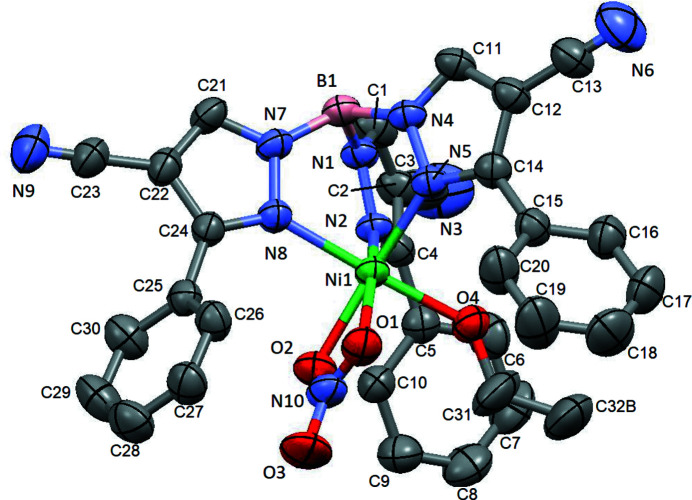
The title compound with displacement ellipsoids drawn at the 50% probability level. H atoms have been omitted for clarity. Only the major component of the Me atom is shown.

Scorpionate, or tris­pyrazolylborate (Tp), ligands were shown early in their existence to readily form octa­hedral sandwich complexes (Tp_2_ *M*) with transition metals (Trofimenko, 1966 ▸; Trofimenko, 1967 ▸). Trofimenko and coworkers reported, in 1987, the synthesis of Tp^Ph^, which they showed was resistant to formation of such complexes due to the bulk of the phenyl substituents (Trofimenko *et al.*, 1987 ▸). Eichhorn and Armstrong showed that this ligand could still form sandwich compounds with increased *M*—N bond lengths (Eichhorn & Armstrong, 1990 ▸). Eichhorn and coworkers later reported the cyano­scorpionates, including the Tp^Ph,4CN^ ligand, for which to date only sandwich compounds have been reported, including those with two borotropic shifted Tp^Ph,4CN^ ligands (Zhao *et al.*, 2007 ▸) and those with one Tp^Ph,4CN^ and one Bp^Ph,4CN^ (bis­pyrazolylborate) ligand (Kadel *et al.*, 2016 ▸). The title Ni^II^ compound (Fig. 1 ▸) is the first reported ‘half-sandwich’ complex of Tp^Ph,4CN^. The Ni atom is coordinated by one Tp^Ph,4CN^ ligand, occupying one face of the pseudo-octa­hedral coordination sphere, one bidentate nitrate ligand and one ethanol ligand. Selected bond distances and angles are given in Table 1 ▸. The Ni—N bond lengths [2.079 (2)–2.103 (2) Å; Table 1 ▸] are similar to those in Tp^Ph,4CN^Bp^Ph,4CN^Ni (Kadel *et al.*, 2016 ▸) and shorter than those in the related full sandwich compound Tp^Ph,Me^_2_Ni (Deb *et al.*, 2012 ▸), in which the steric inter­actions between the phenyl substituents on the two ligands require the ligands to pull away from the metal. The coordination sphere bond angles are as expected for an octa­hedral complex involving a bidentate nitrate ligand, with only the in-plane angles involving the nitrate [O—Ni—O = 61.98 (8), N—Ni—O = 100.89 (8) and 106.86 (8)°] deviating significantly from ideal octa­hedral values. The phenyl rings are rotated such that they are relatively parallel to the other ligands, with dihedral angles between the C—C—O plane of the ethanol ligand and the two phenyl rings surrounding it of 17.278 (7) and 339.433 (16)°, and between the plane of the nitrate ligand and the adjacent phenyl ring of 19.578 (7)°. This results in dihedral angles between the phenyl rings and the pyrazole rings to which they are attached of 51.981 (11) and 52.528 (11)° for the groups surrounding the ethanol ligand and 62.302 (13)° for that adjacent to the nitrate, which are normal for a phenyl­pyrazole moiety and allow for minimization of the inter­action between the *ortho*-H on the phenyl ring and the 4-substituent (or H) on the pyrazole. Full sandwich compounds, because of the need to alleviate inter-ligand inter­actions, are forced to have smaller Ph/pz angles, as evidenced by those in Tp^Ph,Me^_2_Ni (12–30°) (Deb *et al.*, 2012 ▸), Tp^Ph^_2_ *M* (*M* = Fe, Mn, Cd; 9–31°; Eichhorn & Armstrong, 1990 ▸; Reger *et al.*, 1995 ▸), and Tp^Ph,4CN^_2_ *M* (*M* = Fe, Co, Mn; 42–53°; Zhao *et al.*, 2007 ▸). An inter­molecular hydrogen-bonding inter­action exists between the ethanol ligand and the CN substituent on one Tp pyrazole ring (Table 2 ▸).

## Synthesis and crystallization

The title compound was synthesized by adding a solution of 0.200 g (0.36 mmol) of potassium tris­(4-cyano-3-phenyl­pyra­zol­yl)hydro­borate (KTp^Ph,4CN^; Zhao *et al.*, 2007 ▸) in 10 ml of acetone dropwise to a solution of Ni(NO_3_)_2_·6H_2_O (0.100 g, 0.36 mmol) in 5 ml of ethanol. After stirring for 5 minutes, the navy blue solution was filtered and the blue precipitate was washed with ethanol. X-ray quality crystals were grown by slow diffusion of ethanol into an aceto­nitrile solution.

## Refinement

Crystal data, data collection and structure refinement details are summarized in Table 3 ▸. The ethanol ligand was modeled with a 0.572 (13)/0.428 (13) disorder of the methyl C atom.

## Supplementary Material

Crystal structure: contains datablock(s) I. DOI: 10.1107/S2414314621006908/bv4040sup1.cif

Structure factors: contains datablock(s) I. DOI: 10.1107/S2414314621006908/bv4040Isup2.hkl

CCDC reference: 2094536

Additional supporting information:  crystallographic information; 3D view; checkCIF report

## Figures and Tables

**Table 1:** Selected geometric parameters (Å, °)

Ni1—O4	2.071 (2)	Ni1—N2	2.079 (2)
Ni1—N5	2.087 (2)	Ni1—O1	2.104 (2)
Ni1—N8	2.103 (2)	Ni1—O2	2.0812 (19)
			
N5—Ni1—O1	106.86 (8)	O2—Ni1—O1	61.98 (8)
N2—Ni1—O2	100.89 (8)		

**Table 2:** Hydrogen-bond geometry (Å, °)

*D*—H⋯*A*	*D*—H	H⋯*A*	*D*⋯*A*	*D*—H⋯*A*
O4—H4⋯N9^i^	0.83 (3)	2.17 (3)	2.999 (4)	178 (3)

Symmetry code: (i) 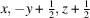 .

**Table 3:** Experimental details

Crystal data	
Chemical formula	[Ni(C_30_H_19_BN_9_)(NO_3_)(C_2_H_6_O)]
*M*_r_	683.14
Crystal system, space group	Orthorhombic, *P* *b* *c* *a*
Temperature (K)	150
*a*, *b*, *c* (Å)	16.627 (6), 18.030 (7), 21.293 (8)
*V* (Å^3^)	6383 (4)
*Z*	8
Radiation type	Mo *K*α
μ (mm^−1^)	0.66
Crystal size (mm)	0.59 × 0.52 × 0.32

Data collection	
Diffractometer	Bruker APEXII CCD
Absorption correction	Numerical (*SADABS*; Bruker, 2013 ▸)
*T*_min_, *T*_max_	0.673, 0.746
No. of measured, independent and observed [*I* > 2σ(*I*)] reflections	118458, 7103, 4543
*R*_int_	0.065
(sin θ/λ)_max_ (Å^−1^)	0.650

Refinement	
*R*[*F* ^2^ > 2σ(*F* ^2^)], *wR*(*F* ^2^), *S*	0.048, 0.146, 1.03
No. of reflections	7103
No. of parameters	448
H-atom treatment	H atoms treated by a mixture of independent and constrained refinement
Δρ_max_, Δρ_min_ (e Å^−3^)	0.76, −0.32

Computer programs: *APEX2* and *SAINT* (Bruker, 2013 ▸), *SIR2004* (Burla *et al.*, 2007 ▸), *SHELXL* (Sheldrick, 2015 ▸) and *OLEX2* (Dolomanov *et al.*, 2009 ▸).

## full crystallographic data

### Crystal data

**Table:**

[Ni(C_30_H_19_BN_9_)(NO_3_)(C_2_H_6_O)]	*D*_x_ = 1.422 Mg m^−^^3^
*M**_r_* = 683.14	Mo *K*α radiation, λ = 0.71073 Å
Orthorhombic, *P**b**c**a*	Cell parameters from 9305 reflections
*a* = 16.627 (6) Å	θ = 3.0–22.8°
*b* = 18.030 (7) Å	µ = 0.66 mm^−^^1^
*c* = 21.293 (8) Å	*T* = 150 K
*V* = 6383 (4) Å^3^	Block, light blue
*Z* = 8	0.59 × 0.52 × 0.32 mm
*F*(000) = 2816	

### Data collection

**Table:**

Bruker APEXII CCD diffractometer	7103 independent reflections
Radiation source: sealed X-ray tube	4543 reflections with *I* > 2σ(*I*)
Graphite monochromator	*R*_int_ = 0.065
Detector resolution: 5.6 pixels mm^-1^	θ_max_ = 27.5°, θ_min_ = 3.7°
φ and ω scans	*h* = −21→20
Absorption correction: numerical (SADABS; Bruker, 2013)	*k* = −23→23
*T*_min_ = 0.673, *T*_max_ = 0.746	*l* = −27→27
118458 measured reflections	

### Refinement

**Table:**

Refinement on *F*^2^	Primary atom site location: structure-invariant direct methods
Least-squares matrix: full	Hydrogen site location: mixed
*R*[*F*^2^ > 2σ(*F*^2^)] = 0.048	H atoms treated by a mixture of independent and constrained refinement
*wR*(*F*^2^) = 0.146	*w* = 1/[σ^2^(*F*_o_^2^) + (0.0804*P*)^2^ + 1.0693*P*] where *P* = (*F*_o_^2^ + 2*F*_c_^2^)/3
*S* = 1.03	(Δ/σ)_max_ = 0.001
7103 reflections	Δρ_max_ = 0.76 e Å^−^^3^
448 parameters	Δρ_min_ = −0.32 e Å^−^^3^
0 restraints	

### Special details

**Table:**

Geometry. All esds (except the esd in the dihedral angle between two l.s. planes) are estimated using the full covariance matrix. The cell esds are taken into account individually in the estimation of esds in distances, angles and torsion angles; correlations between esds in cell parameters are only used when they are defined by crystal symmetry. An approximate (isotropic) treatment of cell esds is used for estimating esds involving l.s. planes.

### Fractional atomic coordinates and isotropic or equivalent isotropic displacement parameters (Å^2^)

**Table:**

	*x*	*y*	*z*	*U*_iso_*/*U*_eq_	Occ. (<1)
Ni1	0.46783 (2)	0.32105 (2)	0.75782 (2)	0.04004 (13)	
O4	0.45090 (12)	0.34845 (12)	0.85134 (10)	0.0554 (5)	
N4	0.59048 (13)	0.20364 (12)	0.75765 (10)	0.0459 (5)	
N7	0.51417 (12)	0.21874 (12)	0.65688 (10)	0.0479 (5)	
N5	0.57845 (12)	0.27386 (11)	0.78134 (10)	0.0455 (5)	
N1	0.44970 (14)	0.15850 (13)	0.75030 (10)	0.0456 (5)	
N8	0.49015 (12)	0.29152 (11)	0.66387 (10)	0.0444 (5)	
N2	0.41089 (12)	0.22004 (11)	0.77343 (10)	0.0435 (5)	
O3	0.38604 (17)	0.50607 (12)	0.71996 (12)	0.0887 (7)	
N10	0.41371 (17)	0.44527 (13)	0.73128 (11)	0.0577 (6)	
C22	0.49747 (15)	0.26308 (16)	0.56234 (13)	0.0511 (7)	
C12	0.68610 (15)	0.22229 (14)	0.82726 (13)	0.0504 (7)	
C21	0.51872 (15)	0.20149 (17)	0.59654 (14)	0.0525 (7)	
H21	0.5340	0.1547	0.5798	0.063*	
C5	0.29602 (14)	0.24381 (15)	0.84526 (13)	0.0482 (6)	
C24	0.48020 (14)	0.31881 (14)	0.60640 (13)	0.0432 (6)	
C2	0.35174 (15)	0.11721 (15)	0.80951 (14)	0.0537 (7)	
C14	0.63667 (14)	0.28587 (14)	0.82355 (12)	0.0458 (6)	
C4	0.35099 (14)	0.19523 (14)	0.80997 (12)	0.0457 (6)	
C11	0.65437 (15)	0.17294 (14)	0.78480 (14)	0.0496 (7)	
H11	0.6747	0.1248	0.7762	0.060*	
N9	0.49338 (19)	0.27259 (18)	0.44209 (14)	0.0826 (8)	
C10	0.25357 (16)	0.30045 (15)	0.81617 (13)	0.0518 (6)	
H10	0.2592	0.3082	0.7723	0.062*	
C1	0.41527 (16)	0.09678 (15)	0.77214 (15)	0.0569 (7)	
H1	0.4318	0.0474	0.7634	0.068*	
C23	0.49527 (19)	0.26954 (18)	0.49556 (16)	0.0611 (8)	
C25	0.45741 (14)	0.39623 (15)	0.59128 (12)	0.0465 (6)	
C15	0.64625 (15)	0.35621 (15)	0.85781 (13)	0.0502 (7)	
C13	0.75612 (18)	0.21118 (17)	0.86449 (15)	0.0632 (8)	
C27	0.48543 (19)	0.52701 (18)	0.58728 (15)	0.0641 (8)	
H27	0.5191	0.5676	0.5981	0.077*	
C28	0.4170 (2)	0.53909 (19)	0.55438 (16)	0.0732 (9)	
H28	0.4024	0.5882	0.5428	0.088*	
C26	0.50660 (17)	0.45578 (16)	0.60520 (13)	0.0541 (7)	
H26	0.5555	0.4478	0.6273	0.065*	
N6	0.81161 (18)	0.20048 (19)	0.89414 (16)	0.0960 (10)	
C3	0.29669 (19)	0.07076 (17)	0.84334 (19)	0.0744 (9)	
C20	0.6497 (2)	0.42321 (17)	0.82685 (16)	0.0735 (9)	
H20	0.6444	0.4242	0.7824	0.088*	
C6	0.28563 (18)	0.23255 (19)	0.90931 (15)	0.0690 (9)	
H6	0.3125	0.1929	0.9299	0.083*	
C16	0.65492 (18)	0.35574 (19)	0.92228 (15)	0.0637 (8)	
H16	0.6536	0.3100	0.9443	0.076*	
N3	0.2532 (2)	0.03462 (18)	0.87100 (18)	0.1132 (12)	
C9	0.20337 (18)	0.34536 (18)	0.85066 (17)	0.0676 (8)	
H9	0.1743	0.3838	0.8302	0.081*	
C30	0.38781 (16)	0.40902 (18)	0.55660 (15)	0.0653 (8)	
H30	0.3535	0.3689	0.5459	0.078*	
C7	0.2353 (2)	0.2801 (2)	0.94312 (17)	0.0846 (10)	
H7	0.2294	0.2737	0.9872	0.102*	
C8	0.1946 (2)	0.3357 (2)	0.91353 (18)	0.0764 (10)	
H8	0.1603	0.3675	0.9368	0.092*	
C19	0.6607 (2)	0.4889 (2)	0.85900 (19)	0.0934 (12)	
H19	0.6635	0.5346	0.8369	0.112*	
C18	0.6677 (2)	0.4876 (2)	0.9229 (2)	0.0931 (12)	
H18	0.6740	0.5327	0.9453	0.112*	
C29	0.36861 (19)	0.4806 (2)	0.53762 (17)	0.0801 (10)	
H29	0.3219	0.4891	0.5130	0.096*	
C17	0.6655 (2)	0.4213 (2)	0.95511 (17)	0.0780 (10)	
H17	0.6712	0.4207	0.9995	0.094*	
B1	0.52890 (17)	0.16769 (17)	0.71325 (17)	0.0480 (7)	
H1A	0.5488	0.1183	0.6987	0.058*	
O1	0.48821 (13)	0.43502 (10)	0.74386 (9)	0.0553 (5)	
O2	0.37070 (11)	0.38682 (11)	0.73135 (9)	0.0562 (5)	
C31	0.4158 (2)	0.4038 (2)	0.88379 (19)	0.0963 (13)	
H31A	0.3580	0.4010	0.8729	0.116*	0.572 (13)
H31B	0.4361	0.4501	0.8643	0.116*	0.572 (13)
H31C	0.3840	0.3799	0.9174	0.116*	0.428 (13)
H31D	0.3766	0.4271	0.8549	0.116*	0.428 (13)
C32A	0.4179 (7)	0.4169 (5)	0.9490 (4)	0.101 (4)	0.572 (13)
H32A	0.3834	0.3808	0.9704	0.152*	0.572 (13)
H32B	0.3986	0.4672	0.9577	0.152*	0.572 (13)
H32C	0.4733	0.4118	0.9642	0.152*	0.572 (13)
C32B	0.4565 (6)	0.4602 (7)	0.9114 (7)	0.099 (5)	0.428 (13)
H32D	0.4744	0.4448	0.9533	0.149*	0.428 (13)
H32E	0.4211	0.5035	0.9152	0.149*	0.428 (13)
H32F	0.5033	0.4732	0.8857	0.149*	0.428 (13)
H4	0.4630 (15)	0.3142 (16)	0.8759 (14)	0.050*	

### Atomic displacement parameters (Å^2^)

**Table:**

	*U*^11^	*U*^22^	*U*^33^	*U*^12^	*U*^13^	*U*^23^
Ni1	0.0524 (2)	0.0270 (2)	0.0407 (2)	0.00113 (13)	−0.00158 (13)	−0.00124 (14)
O4	0.0763 (12)	0.0436 (12)	0.0462 (12)	0.0038 (10)	0.0028 (10)	−0.0018 (10)
N4	0.0548 (11)	0.0306 (12)	0.0522 (14)	0.0034 (10)	−0.0021 (10)	−0.0030 (10)
N7	0.0626 (12)	0.0351 (12)	0.0458 (14)	0.0041 (10)	0.0011 (10)	−0.0070 (11)
N5	0.0535 (11)	0.0338 (13)	0.0492 (13)	0.0022 (9)	−0.0025 (10)	−0.0026 (10)
N1	0.0547 (11)	0.0284 (12)	0.0538 (15)	0.0047 (10)	−0.0050 (9)	−0.0038 (10)
N8	0.0580 (11)	0.0345 (12)	0.0407 (13)	0.0032 (10)	−0.0022 (10)	−0.0013 (10)
N2	0.0531 (11)	0.0294 (12)	0.0480 (13)	0.0044 (9)	−0.0024 (10)	−0.0051 (10)
O3	0.135 (2)	0.0379 (13)	0.0930 (17)	0.0297 (13)	−0.0026 (15)	0.0070 (12)
N10	0.0906 (18)	0.0320 (14)	0.0506 (15)	0.0094 (13)	0.0056 (12)	0.0011 (11)
C22	0.0545 (14)	0.0569 (19)	0.0418 (16)	−0.0010 (13)	−0.0016 (12)	−0.0085 (14)
C12	0.0508 (13)	0.0425 (16)	0.0579 (18)	0.0007 (12)	−0.0047 (12)	0.0049 (14)
C21	0.0626 (15)	0.0471 (17)	0.0478 (18)	0.0010 (13)	0.0021 (12)	−0.0143 (14)
C5	0.0495 (13)	0.0447 (16)	0.0505 (17)	−0.0061 (11)	−0.0008 (12)	0.0010 (13)
C24	0.0470 (12)	0.0440 (16)	0.0385 (15)	−0.0008 (10)	−0.0007 (10)	−0.0017 (12)
C2	0.0584 (15)	0.0334 (15)	0.069 (2)	−0.0043 (12)	−0.0035 (13)	0.0065 (14)
C14	0.0519 (13)	0.0370 (15)	0.0484 (16)	−0.0045 (11)	−0.0010 (12)	0.0020 (13)
C4	0.0496 (13)	0.0393 (16)	0.0482 (16)	−0.0003 (11)	−0.0083 (12)	0.0026 (12)
C11	0.0512 (13)	0.0364 (15)	0.0614 (18)	0.0037 (11)	−0.0010 (13)	0.0042 (13)
N9	0.108 (2)	0.090 (2)	0.0493 (18)	0.0042 (17)	−0.0003 (16)	−0.0112 (16)
C10	0.0562 (14)	0.0512 (17)	0.0481 (16)	0.0014 (13)	−0.0005 (12)	−0.0046 (13)
C1	0.0584 (15)	0.0320 (16)	0.080 (2)	−0.0033 (12)	−0.0101 (14)	0.0009 (14)
C23	0.0725 (18)	0.061 (2)	0.050 (2)	0.0016 (15)	0.0000 (15)	−0.0106 (16)
C25	0.0539 (13)	0.0471 (17)	0.0384 (15)	0.0024 (12)	0.0038 (11)	0.0017 (12)
C15	0.0536 (13)	0.0453 (17)	0.0516 (18)	−0.0026 (12)	−0.0090 (12)	−0.0001 (14)
C13	0.0588 (15)	0.0551 (19)	0.076 (2)	0.0036 (14)	−0.0108 (15)	0.0059 (16)
C27	0.085 (2)	0.0492 (19)	0.057 (2)	−0.0017 (15)	−0.0079 (16)	0.0061 (15)
C28	0.089 (2)	0.057 (2)	0.073 (2)	0.0104 (18)	0.0012 (18)	0.0193 (17)
C26	0.0648 (15)	0.0495 (18)	0.0481 (17)	−0.0026 (13)	−0.0068 (13)	0.0064 (14)
N6	0.0773 (18)	0.094 (2)	0.116 (3)	0.0064 (17)	−0.0359 (18)	0.004 (2)
C3	0.0740 (19)	0.0429 (19)	0.106 (3)	−0.0052 (15)	0.0088 (19)	0.0059 (19)
C20	0.105 (2)	0.049 (2)	0.066 (2)	−0.0111 (17)	−0.0229 (18)	0.0021 (17)
C6	0.0778 (19)	0.069 (2)	0.060 (2)	−0.0040 (16)	0.0055 (16)	0.0170 (17)
C16	0.0732 (18)	0.062 (2)	0.056 (2)	−0.0017 (15)	−0.0090 (15)	−0.0040 (16)
N3	0.112 (2)	0.068 (2)	0.160 (4)	−0.0303 (19)	0.036 (2)	0.015 (2)
C9	0.0640 (17)	0.066 (2)	0.072 (2)	0.0116 (15)	0.0034 (16)	−0.0066 (18)
C30	0.0590 (16)	0.063 (2)	0.074 (2)	−0.0024 (14)	−0.0077 (15)	0.0116 (17)
C7	0.092 (2)	0.105 (3)	0.056 (2)	−0.014 (2)	0.0274 (19)	−0.004 (2)
C8	0.0664 (18)	0.084 (3)	0.078 (3)	0.0008 (17)	0.0190 (18)	−0.015 (2)
C19	0.137 (3)	0.053 (2)	0.090 (3)	−0.014 (2)	−0.037 (2)	−0.006 (2)
C18	0.121 (3)	0.062 (3)	0.097 (3)	−0.004 (2)	−0.026 (2)	−0.029 (2)
C29	0.0684 (18)	0.087 (3)	0.086 (3)	0.0123 (18)	−0.0122 (17)	0.031 (2)
C17	0.094 (2)	0.082 (3)	0.059 (2)	−0.0029 (19)	−0.0154 (17)	−0.021 (2)
B1	0.0575 (16)	0.0351 (18)	0.051 (2)	0.0062 (13)	−0.0023 (14)	−0.0067 (15)
O1	0.0778 (13)	0.0328 (11)	0.0553 (13)	−0.0067 (10)	−0.0019 (9)	0.0011 (9)
O2	0.0644 (11)	0.0387 (11)	0.0657 (13)	0.0056 (9)	−0.0004 (9)	0.0015 (9)
C31	0.107 (3)	0.105 (3)	0.077 (3)	0.005 (2)	0.006 (2)	−0.048 (2)
C32A	0.158 (8)	0.076 (6)	0.071 (5)	0.015 (6)	0.006 (5)	−0.030 (4)
C32B	0.085 (6)	0.081 (8)	0.131 (12)	−0.003 (5)	−0.013 (6)	−0.045 (8)

### Geometric parameters (Å, º)

**Table:**

Ni1—O4	2.071 (2)	C25—C30	1.392 (4)
Ni1—N5	2.087 (2)	C15—C20	1.377 (4)
Ni1—N8	2.103 (2)	C15—C16	1.380 (4)
Ni1—N2	2.079 (2)	C13—N6	1.134 (4)
Ni1—O1	2.104 (2)	C27—H27	0.9500
Ni1—O2	2.0812 (19)	C27—C28	1.354 (4)
O4—C31	1.347 (4)	C27—C26	1.385 (4)
O4—H4	0.83 (3)	C28—H28	0.9500
N4—N5	1.378 (3)	C28—C29	1.374 (5)
N4—C11	1.330 (3)	C26—H26	0.9500
N4—B1	1.537 (4)	C3—N3	1.137 (4)
N7—N8	1.380 (3)	C20—H20	0.9500
N7—C21	1.324 (3)	C20—C19	1.380 (4)
N7—B1	1.532 (4)	C6—H6	0.9500
N5—C14	1.339 (3)	C6—C7	1.398 (5)
N1—N2	1.375 (3)	C16—H16	0.9500
N1—C1	1.335 (4)	C16—C17	1.385 (4)
N1—B1	1.544 (4)	C9—H9	0.9500
N8—C24	1.329 (3)	C9—C8	1.358 (5)
N2—C4	1.341 (3)	C30—H30	0.9500
O3—N10	1.213 (3)	C30—C29	1.389 (4)
N10—O1	1.281 (3)	C7—H7	0.9500
N10—O2	1.274 (3)	C7—C8	1.362 (5)
C22—C21	1.374 (4)	C8—H8	0.9500
C22—C24	1.404 (4)	C19—H19	0.9500
C22—C23	1.427 (4)	C19—C18	1.365 (5)
C12—C14	1.413 (3)	C18—H18	0.9500
C12—C11	1.374 (4)	C18—C17	1.378 (5)
C12—C13	1.423 (4)	C29—H29	0.9500
C21—H21	0.9500	C17—H17	0.9500
C5—C4	1.472 (4)	B1—H1A	1.0000
C5—C10	1.387 (4)	C31—H31A	0.9900
C5—C6	1.390 (4)	C31—H31B	0.9900
C24—C25	1.482 (4)	C31—H31C	0.9900
C2—C4	1.407 (4)	C31—H31D	0.9900
C2—C1	1.373 (4)	C31—C32A	1.409 (8)
C2—C3	1.435 (4)	C31—C32B	1.355 (10)
C14—C15	1.472 (4)	C32A—H32A	0.9800
C11—H11	0.9500	C32A—H32B	0.9800
N9—C23	1.140 (4)	C32A—H32C	0.9800
C10—H10	0.9500	C32B—H32D	0.9800
C10—C9	1.376 (4)	C32B—H32E	0.9800
C1—H1	0.9500	C32B—H32F	0.9800
C25—C26	1.382 (4)		
			
O4—Ni1—N5	89.22 (9)	C16—C15—C14	119.9 (3)
O4—Ni1—N8	177.46 (8)	N6—C13—C12	178.3 (3)
O4—Ni1—N2	89.62 (8)	C28—C27—H27	119.8
O4—Ni1—O1	85.68 (8)	C28—C27—C26	120.3 (3)
O4—Ni1—O2	91.09 (8)	C26—C27—H27	119.8
N5—Ni1—N8	88.25 (8)	C27—C28—H28	119.9
N5—Ni1—O1	106.86 (8)	C27—C28—C29	120.2 (3)
N8—Ni1—O1	94.85 (8)	C29—C28—H28	119.9
N2—Ni1—N5	90.33 (8)	C25—C26—C27	120.7 (3)
N2—Ni1—N8	90.61 (8)	C25—C26—H26	119.6
N2—Ni1—O1	162.07 (8)	C27—C26—H26	119.6
N2—Ni1—O2	100.89 (8)	N3—C3—C2	178.9 (4)
O2—Ni1—N5	168.77 (8)	C15—C20—H20	119.3
O2—Ni1—N8	91.36 (8)	C15—C20—C19	121.4 (3)
O2—Ni1—O1	61.98 (8)	C19—C20—H20	119.3
Ni1—O4—H4	113 (2)	C5—C6—H6	120.3
C31—O4—Ni1	136.8 (2)	C5—C6—C7	119.3 (3)
C31—O4—H4	109 (2)	C7—C6—H6	120.3
N5—N4—B1	121.1 (2)	C15—C16—H16	119.7
C11—N4—N5	109.8 (2)	C15—C16—C17	120.7 (3)
C11—N4—B1	128.6 (2)	C17—C16—H16	119.7
N8—N7—B1	122.2 (2)	C10—C9—H9	119.5
C21—N7—N8	110.2 (2)	C8—C9—C10	121.0 (3)
C21—N7—B1	127.6 (2)	C8—C9—H9	119.5
N4—N5—Ni1	114.50 (15)	C25—C30—H30	120.0
C14—N5—Ni1	137.08 (18)	C29—C30—C25	120.0 (3)
C14—N5—N4	106.83 (19)	C29—C30—H30	120.0
N2—N1—B1	119.8 (2)	C6—C7—H7	119.7
C1—N1—N2	110.3 (2)	C8—C7—C6	120.7 (3)
C1—N1—B1	129.3 (2)	C8—C7—H7	119.7
N7—N8—Ni1	113.23 (16)	C9—C8—C7	119.8 (3)
C24—N8—Ni1	139.46 (18)	C9—C8—H8	120.1
C24—N8—N7	106.8 (2)	C7—C8—H8	120.1
N1—N2—Ni1	115.84 (17)	C20—C19—H19	120.3
C4—N2—Ni1	136.33 (17)	C18—C19—C20	119.4 (4)
C4—N2—N1	106.7 (2)	C18—C19—H19	120.3
O3—N10—O1	122.6 (3)	C19—C18—H18	119.7
O3—N10—O2	122.3 (3)	C19—C18—C17	120.6 (3)
O2—N10—O1	115.0 (2)	C17—C18—H18	119.7
C21—C22—C24	106.1 (2)	C28—C29—C30	120.2 (3)
C21—C22—C23	126.9 (3)	C28—C29—H29	119.9
C24—C22—C23	127.0 (3)	C30—C29—H29	119.9
C14—C12—C13	128.4 (3)	C16—C17—H17	120.3
C11—C12—C14	105.4 (2)	C18—C17—C16	119.5 (3)
C11—C12—C13	126.1 (3)	C18—C17—H17	120.3
N7—C21—C22	108.0 (3)	N4—B1—N1	107.4 (3)
N7—C21—H21	126.0	N4—B1—H1A	110.2
C22—C21—H21	126.0	N7—B1—N4	109.6 (2)
C10—C5—C4	121.7 (2)	N7—B1—N1	109.2 (2)
C10—C5—C6	118.8 (3)	N7—B1—H1A	110.2
C6—C5—C4	119.4 (3)	N1—B1—H1A	110.2
N8—C24—C22	108.9 (2)	N10—O1—Ni1	90.84 (15)
N8—C24—C25	125.5 (2)	N10—O2—Ni1	92.07 (16)
C22—C24—C25	125.5 (2)	O4—C31—H31A	105.2
C4—C2—C3	125.1 (3)	O4—C31—H31B	105.2
C1—C2—C4	106.2 (2)	O4—C31—H31C	106.3
C1—C2—C3	128.7 (3)	O4—C31—H31D	106.3
N5—C14—C12	109.1 (2)	O4—C31—C32A	128.3 (5)
N5—C14—C15	123.4 (2)	O4—C31—C32B	124.3 (6)
C12—C14—C15	127.5 (2)	H31A—C31—H31B	105.9
N2—C4—C5	124.0 (2)	H31C—C31—H31D	106.4
N2—C4—C2	108.9 (2)	C32A—C31—H31A	105.2
C2—C4—C5	127.2 (2)	C32A—C31—H31B	105.2
N4—C11—C12	108.9 (2)	C32B—C31—H31C	106.3
N4—C11—H11	125.6	C32B—C31—H31D	106.3
C12—C11—H11	125.6	C31—C32A—H32A	109.5
C5—C10—H10	119.9	C31—C32A—H32B	109.5
C9—C10—C5	120.2 (3)	C31—C32A—H32C	109.5
C9—C10—H10	119.9	H32A—C32A—H32B	109.5
N1—C1—C2	107.9 (2)	H32A—C32A—H32C	109.5
N1—C1—H1	126.0	H32B—C32A—H32C	109.5
C2—C1—H1	126.0	C31—C32B—H32D	109.5
N9—C23—C22	178.1 (4)	C31—C32B—H32E	109.5
C26—C25—C24	122.3 (2)	C31—C32B—H32F	109.5
C26—C25—C30	118.5 (3)	H32D—C32B—H32E	109.5
C30—C25—C24	118.9 (2)	H32D—C32B—H32F	109.5
C20—C15—C14	121.5 (3)	H32E—C32B—H32F	109.5
C20—C15—C16	118.5 (3)		

### Hydrogen-bond geometry (Å, º)

**Table:**

*D*—H···*A*	*D*—H	H···*A*	*D*···*A*	*D*—H···*A*
O4—H4···N9^i^	0.83 (3)	2.17 (3)	2.999 (4)	178 (3)

Symmetry code: (i) *x*, −*y*+1/2, *z*+1/2.
